# If Motivation Was a Key Factor in Aerobic Performance in Tropical Climate?

**DOI:** 10.3389/fpsyg.2020.619198

**Published:** 2021-02-01

**Authors:** Guillaume R. Coudevylle, Stéphane Sinnapah, Aurélie Collado, Fabien Fenouillet, Olivier Hue, Matthieu Parrat, Nicolas Robin

**Affiliations:** ^1^Laboratory ACTES (EA 3596), University of the French Antilles, Pointe-à-Pitre, France; ^2^Laboratoire Interdisciplinaire en Neurosciences, Physiologie et Psychologie: Apprentissages, Activité Physique, Santé (LINP2-2APS), University of Paris Nanterre, Nanterre, France

**Keywords:** motivation, environmental stress, mental abilities, aerobic performance, hot-wet climate

## Abstract

This mini review examines the impact of tropical climate (TC) on motivational factors during aerobic performance and proposes the tracks of an integrative theoretical model to better understand the direct and indirect motivational mechanisms that can operate on athletic performances. TC is detrimental for aerobic performance and, although it clearly induces physiological constraints, these do not seem to be the only factors that explain the performance decline. Indeed, TC performance researchers have developed a theory of anticipation, which suggests that the brain commands a reduction in effort to protect the body from probable harm and heatstroke risk. The objective of this mini review is thus to examine the possibility that motivation may be a key factor in TC performance. The main psychological impacts of TC on aerobic performance are reviewed and an integrative theoretical model is presented that may help to better understand the mechanisms of motivation.

## Introduction

The hot and wet conditions of the tropical climate (TC) have a negative impact on endurance exercise (Hue, 2011). According to the Köppen Climate Classification, the TC is characterized by warm temperatures and fully humid and hot summers, with an average temperature of 27.4°C (81.3°F). The high level of humidity, which impedes efficient sweat evaporation, has a negative impact on performance by reducing thermolysis processes (Galloway and Maughan, 1997). This climate can undermine thermoregulation (Nybo, 2010), which may then unbalance the hydration status (Stuempfle et al., 2011) by compromising the usefulness of sweating (Brotherhood, 2008). Yet, the core temperature reached by athletes during exercise in TC is not thought to fully explain the extent of the drop in performance. Aerobic performance is reduced by TC (e.g., shorter distance in time trials and increased time to cover a distance), but its physiological impacts do not fully explain the decline in performance, as some studies have demonstrated that the greater performances in runners elicited higher core temperature increases and higher water losses (Noakes, 2007).

An alternative explanation can be glimpsed in the “central governor model” theory (Noakes, 2012). According to this theory, the brain plays the role of an effort regulator, anticipating and adapting instantaneously and continuously to ensure the effort required before the rupture of the physiological balance in the body. Thus, psychological parameters may come into play in the performance declines (see Coudevylle et al., 2019). The studies thus far that have focused on psychological parameters have mainly been oriented towards cognitive processes (e.g., reaction time, memory, and attention), perceptions (e.g., RPE, thermal comfort), or affect (see Robin et al., in press). Yet like the physiological factors, these psychological factors seem to be insufficient to fully explain the decrease in long-duration exercise performance. Given the lack of a satisfactory explanation, the hypothesis of an anticipatory strategy has thus also been considered, according to which athletes end their efforts despite being in a position to continue them, with the assumption being that they do so to avoid the probable subsequent occurrence of an endangerment of the organism (see Tucker et al., 2006). To our knowledge, no empirical studies have focused on motivational factors in athletes in relation to the environmental climate, and yet the hypothesis of motivational involvement is plausible. According to Vallerand and Thill (1993), “the concept of motivation represents the hypothetical construct used to describe the internal and/or external forces producing the initiation, direction, intensity and persistence of the behavior” (p. 18). Thus, this article attempts to describe the motivational mechanisms that might be involved in the decline in aerobic performance in TC and to determine if actual motivational models would be relevant to fully explain the deleterious impact of TC on performance. First, this mini review presents the theoretical background concerning the psychological impacts of TC. Second, it presents an integrative theoretical model that might provide a better understanding of the motivational mechanisms.

To achieve these objectives, citations from *PubMed Central* were identified from the earliest record up to December 2020 using the following Boolean phrase: “tropical climate” OR “hot and wet climate” OR “subtropical” AND “motivation” AND “aerobic performance” AND “sport” AND “effort”. To be included, the studies conducted in TC had to have ambient temperatures around 31°C and rH ≥ 50%. Of the 55 occurrences obtained, all studies without any link to or involvement in competitive physical activities, exercise or sport performance were excluded. All studies that provided theoretical support in line with the objective of this study as a theoretical model to understand the issue of motivation for aerobic performance under heat stress conditions were included.

## Psychological Impacts of Tropical Climate on Athletes During Aerobic Performance

### Consequences of TC for Cognitive Performance

Performance in certain “aerobic” activities (e.g., trail running) requires a high level of cognitive functioning. Depending on their complexity, cognitive tasks may be differently impacted by TC (Robin et al., in press). Whereas heat stress does not affect tasks like simple tasks, more complex tasks such as a pointing task (Ernwein and Keller, 1998; Robin et al., 2017) or sustained attention (Vasmatzidis et al., 2002; Coudevylle et al., 2018) and working memory (Gaoua et al., 2018) tasks are negatively affected. The effects of heat or TC on cognitive performance depend on multiple personal factors − such as level of expertise, sex, hydration status, and heat acclimation − and many external factors, including task duration, the methodology utilized to attain hyperthermia, and the intensity and duration of the thermal stressor (for a review, see Gaoua, 2010; Schmit et al., 2017). In the heat, more cognitive resources is needed to remain focused on an attention task (Chase et al., 2003) as human cognitive capacities are limited (Kahneman, 1973; Gaoua, 2010). We can assume that the discomfort induced by heat induces a cognitive cost (Robin et al., in press) and thus that all cognitive resources are not available for the performance of the sport task.

### Consequences of TC for Psychological Perception

Subjective thermal measures (e.g., thermal comfort and sensation) can be considered psychological markers of the influence of TC. These markers appear to be mediating variables between objective heat, hygrometry and factors of a psychological nature (e.g., at the cognitive and motivational levels). Other studies have shown that high relative humidity reduces the perceived thermal comfort (e.g., Lan et al., 2008). Causal factors for individual differences have been investigated (e.g., sex, age, and circadian rhythm), but no clear and consistent conclusions as to the significance and size of the inter-group differences in thermal comfort have been reached (Wang et al., 2018).

Another psychological marker of the influence of TC, particularly during exercise, is the rating of perceived exertion (RPE; Borg, 1962) and it seems interesting to address athletes’ underlying representations of their pain in relation to health. Several studies have suggested that a high level of motivation when exerting oneself under heat stress can lead to ignoring the early signs and symptoms of a heat-related disability (Parsons et al., 2019). Even if this has not been demonstrated, it is possible that the thermal discomfort felt by athletes could be reduced or, on the contrary, increased according to their representations of the risks they think they are taking with their health by continuing the effort. Two athletes can perceive thermal discomfort and exertion in the same way, but one may think that there is a risk for health and decide to stop, while the other does not and chooses to continue. Finally, the feeling of fatigue appears to occur before any damage to body systems, and it is common to see the term “volitional fatigue”, indicating that subjects have decided to reduce intensity or stop exercising (Cordeiro et al., 2017).

### Consequences of TC for Affective States

Affective states, as measured by the Positive and Negative Affect Schedule (PANAS; Watson et al., 1988) or the Feeling Scale (Hardy and Rejeski, 1989), may be another psychological markers for observing the impact of TC on athletes and determining whether or not intervention strategies are effective. While Gaoua et al. (2012) showed that participants reported higher negative affect scores in a hot and dry environment than in neutral condition, other studies (Coudevylle et al., 2018; Robin et al., in press) showed that TC reduced positive affect scores without influencing negative affect. According to Acevedo and Ekkekakis (2001), the impact of the increase in the environmental temperature on psychological factors often precedes physiological deterioration. Thus, examining the role played by both affect and mood states in the link between thermal and effort perceptions and the ability to remain motivated to achieve goals could be an interesting avenue of research.

### The Anticipatory Strategy Hypothesis

During long-distance races, several observations such as the fact that some athletes increase their running pace as they approach the finish line or that they finish these races without evidence of catastrophic homeostasis failure suggest that individuals may reduce their effort as a way to anticipate and avoid the occurrence of danger to the organism (Noakes, 2007). The idea is that the central nervous system (CNS) seems to regulate exercise intensity in order to limit excessive hyperthermia (Marino, 2004). Central temperature increases during exercise but does so to the same extent in neutral and tropical situations (Tucker et al., 2004). When a certain RPE is reached, athletes usually reduce the exercise intensity, but this decrease occurs earlier in TC than in neutral climate (Hue et al., 2019). In other words, people lock in at an intensity acceptable to them that corresponds to their comfort zone. When the acceptable RPE is exceeded, they then lower the intensity even though there are no physiological reasons to do so (neither thermal nor hydric stress). The brain may thus cause a reduction in intensity or a cessation of effort to maintain thermal homeostasis. It is interesting to note that the rate of induced internal heat build-up allows for an anticipated reduction in the intensity of exercise to a fixed level of perceived effort (i.e., before the physiological constraints arrive) during a long race or aerobic exercise. This occurs earlier and more significantly in TC than in neutral and cold environments (Tucker et al., 2006).

This approach is interesting but, as it has been envisaged up to now, the phenomenon is reduced to its neurological component and the possible psychological corollaries are not considered. Yet, if motivation is defined as the energy with which we are inclined to carry out an action, then it can be assumed that other explanations, such as the theories of motivation, should be considered.

## Motivation for Aerobic Exercise in Tropical Climate

### The Theoretical Models That Can Explain Motivation in Tropical Climate

Very few studies to our knowledge have examined the links between motivational factors and exercise or athletic performance in TC. For instance, Craig (2003) indicated that perceptions play on the “emotion/motivation” complex. The ingestion of menthol as a cooling technique illustrates this type of relationship. Menthol does not reduce body or skin temperature in athletes (Barwood et al., 2015), but it does stimulate cold receptors (Cheung, 2010) and induces a sensation of coolness (Mündel and Jones, 2010), which alters thermal perceptions. Thus, the use of a psychological technique could favorably influence the motivation to maintain an effort. Although not yet clearly examined, the effects of TC on motivational factors would be interesting to assess as these factors are related to the previously reported psychological responses. While the factors previously evoked (e.g., cognitive performance, thermal perceptions, and affect) have been clearly identified in the literature, very little is known about the role played by motivational factors (e.g., self-confidence, and value of the task). If an athlete’s performance is less than the critical physiological values would lead us to expect, motivational explanations should be considered. The question is how motivation could be involved in the decline of aerobic performance in TC.

There is no theoretical model that directly addresses motivational issues related to heat stress. However, by examining the role of cognitive appraisal in understanding the body’s response to physical exertion in environmentally stressful conditions, the transactional psychobiological model from Acevedo and Ekkekakis (2001) could potentially permit to study aerobic exercise in TC. In their model, the authors presented the influence of psychological mediators (e.g., personality, attitude, motivation, and past experience) on the threat/challenge process through effort sense, affect and thermal sensation. It is interesting to note that, according to this model, motivation appears upstream of perceptions and affect and that this two-phase (threat/challenge) approach is found in other theories. Many theorists have adopted the idea that motivation responds to such an intrinsic biphasic organization (Schneirla, 1959; Elliot, 2008). The brain is thus assumed to have two major motivational systems, one appetitive and the other aversive or defensive, and each varies in terms of activation or excitement. Excitement is considered to represent the intensity of activation (metabolic and neural) of the appetitive or aversive system, or the coactivation of the two systems (see Cacioppo and Berntson, 1994). In parallel, Saunders et al. (2018) explained that the dopaminergic system, which is the source of motivation, functions in a Pavlovian fashion (i.e., it is strengthened with reward and achievement). Thus, if we compare this with the coactivation model of Cacioppo and Berntson (1994), there is an overactivation of the appetitive system compared to the aversive system. It can then be assumed that producing aerobic effort in a TC can reduce or stop the arrival of dopamine, as exercise continues, thereby leading to negative emotions and aversive or avoidance reactions such as early cessation of exercise to stop physical and thermal discomfort.

Among the many models of motivation, the psychobiological model of endurance performance (Pageaux, 2014) may offer an integrating framework for understanding the problems of long-duration races in the heat. This effort-based decision-making model explains self-paced endurance performance on the basis of the motivational intensity theory (Brehm and Self, 1989). It postulates that the conscious regulation of running rhythm is determined primarily by the following five cognitive/motivational factors: (1) perception of effort (2) potential motivation (3) knowledge of the distance in relation to the time to travel it (4) knowledge of the distance to the time remaining to travel, and (5) previous experience/memory of perceived exertion during exercise of varying intensity and duration. The thermal comfort could be a sixth factor with the particularity of influencing other factors such as the perception of effort and potential motivation. This variable, measured in numerous studies of environmental conditions (see Candas and Dufour, 2005), could be integrated to the psychobiological model of endurance performance to examine the psychological consequences of performance in aerobic events.

Thus, there is no theoretical model that integrates within the same structure all the factors – internal, external, and their combinations − that influence the motivation to continue producing aerobic efforts in TC. Therefore, integrating the elements from different models could be a promising approach.

### An Integrative Model of Motivation for Aerobic Exercise in Tropical Climate

**Figure 1:**
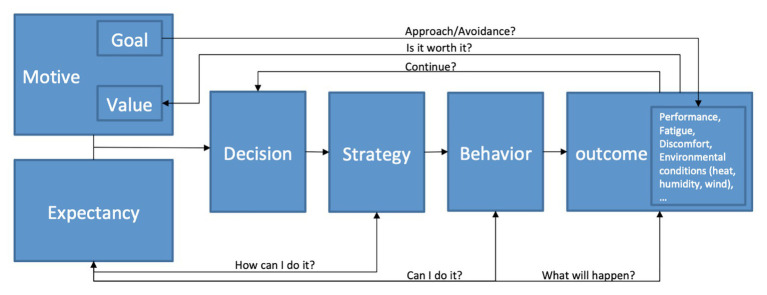
Integrative model of motivation for aerobic performance in tropical climate.

An integrating framework to understand the motivational process of long-duration races in heat stress situations would be to combine several theoretical models. With this in mind, the theoretical models of Heckhausen and Heckhausen (2008) and Fenouillet (2016) considering motivation as a dynamic process could serve as a basis for this theoretical meta-model. This conception allows us to estimate that the initial motivational state will evolve according to the difficulties that the athlete will encounter. The different modules, as well as the relationships between them, make it possible to specify the different factors evoked in this article (see Figure 1).

“Motive” represents the “potential motivation” described in the Pageaux’s (2014) model. Among the motivations, the goals are understood in the sense of approach (e.g., to finish the race, to obtain a good classification, to apply my strategy) and avoidance (e.g., to suffer pain, heat, to lose the race, and not to apply my racing strategies) goals as defined by model of Cacioppo and Berntson (1994), Elliot (2008) or Saunders et al. (2018). Indeed, according to Cacioppo and Berntson (1994) and Saunders et al. (2018), the brain has two major motivational systems, one appetitive, producing dopamine and exercise maintenance behaviors, and the other aversive, producing cortisol and defensive behaviors, each varying in terms of activation or arousal. In other words, excitation is not considered here to have a distinct substrate, but rather to represent the intensity of activation (metabolic and neural) of the appetitive or aversive system or the coactivation of both.

Several models (Lewin et al., 1944; Atkinson, 1964; Vroom, 1964) allow us to say that there is a strong relationship between the expectation and the value of an activity. The success of an easy activity has less value than the success of a difficult activity (Eccles and Wigfield, 2002). Motivation is the product of success expectations and task value and if one of the two factors is lower, motivation can be lower. According to Kukla (1972), the individual re-evaluates his or her expectations and the value assigned to the task for each result or perceived difficulty. In Pageaux’s (2014) model, there are “knowledge of the distance to the time remaining to travel” and “previous experience/memory of perceived exertion during exercise of varying intensity and duration.” The role of beliefs about self-efficacy (Bandura, 1997) or about expectancy and the value of the task (Kukla, 1972) provide theoretical input for understanding the cessation of effort not only at the end of the race but also throughout the event. In long-duration events, athletes regularly re-evaluate and beliefs change according to their perceptions and the context (i.e., presence of other participants, public, and being close to the goal). These beliefs then play a role in whether or not physical effort and concentration are maintained, with all the consequences that this may have on perceptions and emotions. Motivation to maintain effort in long-duration physical events in TC seems to be a dynamic process in perpetual self-regulation.

The “decision” to act is essential in order to distinguish the initial motivation part from the volition part, which will better explain the persistence of the action (Heckhausen and Heckhausen, 2008; Fenouillet, 2016). Once the individual has decided to act, the individual’s logic is no longer to weigh the pluses and minuses in order to take action but rather decisions that are linked to the action taken, such as whether or not the effort should be maintained given the perception of environmental conditions. The “strategic aspect” that comes next allows referring to the strategies that the athlete will put in place before engaging in the action such as the use of menthol to reduce the feeling of heat or the use of a mental technique. “Behavior” refers to the activity itself, which in this case is running. During the activity, the individual will have a perception of the effort, which is likely to act directly on the behavior. Finally, the “outcomes” are of various kinds. There is the performance achieved, but also the perception of fatigue and heat, which will then serve as a basis for making decisions on whether or not to maintain the activity.

External factors not shown on the model may influence the different modules and explain the relationships between them. Staying highly focused on behavior with some strategy (on running tactics, hydration/feeding, cooling, music, or positive motivational self-talk, for examples) would allow the athlete to pay less attention to pain, fatigue, and/or thermal discomfort. Since the perceptions of pain and thermal discomfort are not greatly increased by this work of concentration, the decrease in positive affect and/or the increase in negative affect would also be slowed down. There is a two-way relationship between expectations and strategies, behavior, or results. Feeling negative perceptions and affect later in the event (and therefore closer to achieving the objective) should reinforce the athlete’s expectations of success (maintaining self-confidence) and the value of the task (pain and discomfort are less than the benefits obtained in the case of victory). The decision to continue the effort would thus be maintained or even increased. Conversely, poor focus on a predetermined objective can push the athlete to pay more attention to pain and/or thermal discomfort. As these perceptions of pain and thermal discomfort are greatly increased, the increase in negative affect could also be accelerated. Experiencing such perceptions and affects early in the event would lead the athlete to re-evaluate his or her expectations of success (decreased self-confidence) and the value of the task (the disadvantages appearing to be more important than the advantages obtained in the case of victory) downwards. The decision to maintain the effort would potentially be compromised. This is a more volitional than motivational approach where strategies are aimed at keeping the main objective afloat no matter what the cost (see Carré and Fenouillet, 2009).

## Conclusion

The theoretical models of Heckhausen and Heckhausen (2008) and Fenouillet (2016) could serve as a basis for a theoretical meta-model to understand the direct and indirect motivational mechanisms that can affect athletic performance. Such meta-model (see Figure 1) could provide the basis for an integrative framework for understanding the motivation of athletes to achieve aerobic performance in particular heat stress conditions such as TC. This proposed model supposes to be tested by empirical studies. Motivation can be improved by taking action at different levels. The search for high-level performance thus implies investigating each of these levels (perception, cognition, affect, representations, and beliefs) and assessing the possible margins of progression for each athlete. However, first and foremost, this type of investigation can only be carried out on the basis of a thorough medical examination. Implementing psychological strategies to motivate athletes to keep up an effort can be extremely dangerous to their health, just as the administration of too much menthol can be (Bain et al., 2015; Coudevylle et al., 2019). The priority of coaches should be to first take into account the athlete’s health and then optimize performance by improving thermal comfort through mental and objective techniques (e.g., pre-, per-, and post-cooling).

Other avenues of research might be interested in how the investigation of “trait” factors (e.g., achievement goals, and explanatory styles) and psychological “states” (e.g., variables related to performance, particularly anxiety, coping, flow, and self-confidence) influence athletes’ abilities to better cope with environment stress during their events.

Finally, more than 33% of the world’s population lives in the tropics, a hot and wet environment in which major sport events like the 2014 Football World Cup and the 2016 Olympic Games have taken place (both events in Brazil). This will be the case in the 2021 Olympic Games in Tokyo, where a mean 30°C and 80% rH are expected. This mini-review will give athletes and their coach’s food for thought to better prepare for such events.

## Author Contributions

All authors contributed to the manuscript redaction, from the plan conception to the review of literature to the corrections. All authors contributed to the article and approved the submitted version.

### Conflict of Interest

The authors declare that the research was conducted in the absence of any commercial or financial relationships that could be construed as a potential conflict of interest. We specify that the first author is one of the co-editors. Another editorial team has therefore handled this paper.
